# Total *Astragalus* saponins can reverse type 2 diabetes mellitus-related intestinal dysbiosis and hepatic insulin resistance *in vivo*

**DOI:** 10.3389/fendo.2023.1190827

**Published:** 2023-11-20

**Authors:** Leilei Ma, Xiaojin La, Biwei Zhang, Wenxuan Xu, Chunyu Tian, Qianru Fu, Meng Wang, Chenxi Wu, Zhen Chen, Hong Chang, Ji-an Li

**Affiliations:** ^1^ School of Public Health, North China University of Science and Technology, Tangshan, China; ^2^ He Bei Key Laboratory of Integrated Traditional Chinese and Western Medicine for Diabetes and Its Complications, College of Traditional Chinese Medicine, North China University of Science and Technology, Tangshan, China; ^3^ Oriental Herbs Korlatolt felelossegu tarsasag, Budapest, Hungary

**Keywords:** type 2 diabetes mellitus, intestinal microbiota, insulin resistance, total *Astragalus* saponins, gut-liver axis

## Abstract

**Objective:**

Intestinal flora homeostasis in rats with type 2 diabetes mellitus (T2DM) was evaluated to explore the effects of total *Astragalus* saponins (TAS) on hepatic insulin resistance (IR).

**Methods:**

Six-week-old male Sprague–Dawley rats were fed high-fat and high-sugar diet for 4 weeks and intraperitoneally injected with streptozotocin to induce T2DM, and they were then randomly divided into control, model, metformin, and TAS groups. Stool, serum, colon, and liver samples were collected after 8 weeks of drug administration for relevant analyses.

**Results:**

TAS reduced fasting blood glucose, 2-hour postprandial blood glucose, area under the curve of oral glucose tolerance test, glycated serum protein, homeostasis model assessment of insulin resistance, total cholesterol, triglyceride, and low-density lipoprotein cholesterol levels in T2DM rats but increased insulin, C-peptide, and high-density lipoprotein cholesterol levels. Moreover, TAS improved the morphology and structure of liver and colon tissues and improved the composition of the intestinal microbiome and bacterial community structure at different taxonomic levels. In addition, TAS increased the protein expression of hepatic IRS-1, PI3K, PDK1, and p-AKT and decreased the protein expression of p-GSK-3β. Meanwhile, TAS increased the mRNA expression of liver PDK1, PI3K, and GS and decreased the mRNA expression of GSK-3β.

**Conclusion:**

TAS can ameliorate T2DM-related abnormal glucose and blood lipid metabolism, intestinal dysbiosis, and IR.

## 1 Introduction

Diabetes mellitus, as a metabolic disease, is an increase in blood glucose levels due to reduced insulin secretion or defective insulin action caused by a variety of etiological factors, including genetics and environment. According to the latest statistics from the International Diabetes Federation, there are about 537 million people with diabetes worldwide, and it is expected that 783 million people will have diabetes worldwide in 2045 (1). China’s diabetes crisis is also approaching, the latest data show that about 140.9 million people in China have diabetes, and it is expected that the number of diabetics in China will reach 174.4 million by 2045 (2). Among them, Type 2 diabetes mellitus (T2DM) accounts for more than 90% of the total number of diabetes patients (3). With the increasing prevalence and societal burden of T2DM (1–3), research on its pathogenesis and prevention has become a major public health concern worldwide.

The liver is one of the key organs that regulate glucolipid metabolism and maintains and regulates glucose homeostasis through glycogen synthesis and gluconeogenesis. Therefore, hepatic insulin resistance (IR) is crucial for the development of T2DM.

The intestinal flora is considered to be a new and complex organ. The intestinal microbiota plays an important role in the metabolism and immunomodulation of the organism (4, 5). Studies have shown that changes in the intestinal microbiome are closely related to the development of metabolic diseases, such as T2DM. Disordered intestinal microbiota leads to abnormal intestinal metabolites, intestinal barrier damage, increased barrier permeability, increased endotoxin and pro-inflammatory cytokine production, and increased energy intake, which can induce IR and ultimately trigger metabolic disorders and chronic inflammatory responses in patients with T2DM (4–7).

Both the intestines and liver originate in the foregut during the embryonic stage, and they are anatomically interconnected by the portal vein, through which approximately 80% of the returning blood from the intestines feeds the liver. Many toxins and intestinal microbiota products are absorbed through the intestine via hepatic metabolism, while bile secretion and enterohepatic circulation via the liver can influence intestinal functions (8, 9). Hence, pathologically imbalanced intestinal microbiota may be an important factor contributing to hepatic IR. Therefore, regulating intestinal microbiome homeostasis is a new therapeutic target to improve hepatic IR.

*Astragalus* is a commonly used herbal medicine in the clinical treatment of T2DM, and total Astragalus saponins (TAS) is one of the main active of *Astragalus* (10). Modern pharmacological studies have shown that TAS has various pharmacological activities, including hypoglycemic, immunomodulatory, antioxidant, multi-organ protection, antiviral, and antitumor proprieties (11, 12). Particularly, it can inhibit lipase and aldose reductase activities and prevent free radical scavenging and nitric oxide release to exert multi-target synergy in diabetes treatment (13). Studies have shown that Astragaloside IV (AS-IV), which is the main monomeric component (approximately 21.3%) of TAS, can regulate glucose and insulin levels, improve blood lipid metabolism, reduce oxidative stress damage in the liver, improve IR, positively regulate the abundance and diversity of intestinal microbiota, and increase butyric acid levels in mice with T2DM (14, 15). The above research results suggest that one of the mechanisms of action of TAS in improving glucose metabolism may be associated with regulating intestinal microbiome homeostasis and improving hepatic IR.

Therefore, this study aimed to investigate the mechanisms underlying the influence of TAS on hepatic IR by observing its effects on glucose metabolism and intestinal microbiota using experimentally induced T2DM *in vivo* models.

## 2 Materials and methods

### 2.1 Materials

TAS (purity: UV ≥98%) was purchased from Shaanxi Xintianyu Biotechnology Co., Ltd. (Shaanxi, China). Streptozotocin was purchased from Sigma (Darmstadt, Germany). Metformin hydrochloride tablets were purchased from Sion-American Shanghai Squibb Pharmaceuticals Ltd (Shanghai, China). Biochemical test kits for glycosylated serum protein (GSP), total cholesterol (TC), triglycerides (TG), low-density lipoprotein cholesterol (LDL-C), and high-density lipoprotein cholesterol (HDL-C) were purchased from Nanjing Jiancheng Bioengineering Institute (Nanjing, China). Increased insulin (INS), C-peptide (C-P), interleukin-1β (IL-1β), and tumor necrosis factor-α (TNF-α) were purchased from Jiangsu Meibiao Biotechnology Co., Ltd. (Jiangsu, China). The primary antibody against β-actin was purchased from ABclonal Technology Co., Ltd. (Wuhan, China). The primary antibodies against insulin receptor substrate 1 (IRS-1), phosphoinositide 3-kinase (PI3K), 3-phosphoinositide-dependent kinase 1 (PDK1), phospho-protein kinase B (p-AKT) and phospho-glycogen synthase kinase-3β (GSK-3β) were purchased from Proteintech Group, Inc. (Wuhan, China). HRP-sheep anti-rabbit IgG + HRP-sheep anti-mouse IgG was purchased from Boster Biological Technology Co., Ltd. (Wuhan, China).

### 2.2 Animals

All procedures conformed to the Guide for the Care and Use of Laboratory Animals published by the National Institutes of Health, and all animal experiments were approved by the Animal Ethics Committee of North China University of Science and Technology (approval No. LX2019084). A total of 40 specific pathogen-free (SPF) male Sprague-Dawley rats (weight: 180 ± 10 g; age: 6 weeks old) were purchased from Beijing HFK Biotechnology Co., Ltd (China; license No. SCXK [jing] 2020-0004, certificate No. 110322200101794713). The animals were housed in SPF clean animal room and provided sufficient water and feed. Acclimatization feeding was performed for 1 week. The animals were maintained at room temperature (22–25°C), 50%–60% humidity and 12 h-light/dark time cycles. Sterilized bedding was changed daily.

### 2.3 *In vivo* T2DM model establishment and drug administration

Eight rats were randomly assigned to the control group (fed normal feed), whereas the remaining 32 rats assigned to the high-fat and high-sugar feed group (protein 20 kcal%, carbohydrate 35 kcal%, fat 45 kcal%) for 28 days. Then, rats in the high glucose and fat group were intraperitoneally injected with streptozotocin (30 mg/kg), and the rats in the control group were intraperitoneally injected with citric acid-sodium citrate buffer. After 72 h, rats with fasting blood glucose (FBG) ≥11.1 mmoL/L or blood glucose ≥16.7mmol/L were considered diabetic (16). Eight experimental rats that were not successfully modeled were not included in the study. The T2DM rats were randomly divided into three groups (*n* = 8 each group), which were intragastrically administered metformin (200 mg/kg/day) (17, 18) and TAS (80 mg/kg/day) (19, 20) for 8 weeks. An equal volume of saline solution was intragastrically administered to the control and model rats.

### 2.4 Anesthesia and sampling

After the drug had been administered, rat feces were collected in enzyme-free lyophilized tubes and stored. The rats were anesthetized via intraperitoneal injection of 10% chloral hydrate-ethyl carbamate (1:1.5 mL/kg). Blood was collected from the abdominal aorta and the rats were then euthanized, and serum was collected for biochemical testing. A portion of the liver and colon tissues were fixed with 4% paraformaldehyde, and the rest of the samples were stored at −80°C until further analysis.

### 2.5 Determination of representative components and contents of TAS by high performance liquid chromatography

High performance liquid chromatography was performed to determine the representative components of the TAS aqueous solution (21). An Eclipse XDB-C18 column (4.6 × 250 mm, 5 μm; Agilent Technologies, Santa Clara, CA, USA) set at 40°C was used. The injection volume was 10 μL for the standard substance and samples, the flow rate was set to 1.0 mL/min, and the ratio of acetonitrile: water was 32:68. An evaporative light-scattering detector was used to quantify the components.

### 2.6 Serum-related biochemical analysis

Rat serum was aliquoted and tested for glycated serum protein (GSP), insulin (INS), C-peptide (C-P), interleukin-1β (IL-1β), tumor necrosis factor-α (TNF-α), total cholesterol (TC), triglycerides (TG), low-density lipoprotein cholesterol (LDL-C), and high-density lipoprotein cholesterol (HDL-C) using commercial kits according to the manufacturer’s instructions. The homeostasis model assessment of insulin resistance (HOMA-IR) was calculated based on the FBG and fasting serum INS results (22).

### 2.7 Hematoxylin-eosin and Periodic Acid Schiff tissue staining

The 4% paraformaldehyde-fixed liver and colon samples were cut to appropriate sizes and placed in an embedding tank under running water overnight. Subsequently, the tissues were dehydrated and embedded in paraffin, cut into sections with a thickness of 5 µm, and stained with Hematoxylin-eosin (HE) and Periodic Acid Schiff (PAS) to observe the morphological changes of the liver and colon tissues.

### 2.8 Fecal genomic DNA extraction and detection

The genomic DNA of the samples was extracted by the CTAB method, and the purity and concentration of the DNA were checked. PCR amplification of selected V3-V4 variable regions was performed using specific primers with barcode and high fidelity DNA polymerase according to the selection of sequencing regions. PCR products were detected by 2% agarose gel electrophoresis, and the target fragments were recovered by gel cutting using AxyPrep DNA Gel Recovery Kit (AXYGEN, USA). Referring to the preliminary quantification results of electrophoresis, the PCR-amplified recovered products were detected and quantified by a QuantiFluor-ST Blue Fluorescence Quantification System (Promega, USA) and mixed in the appropriate ratio according to the sequencing volume requirement of each sample. Library construction was performed using the NEB Next Ultra DNA Library Prep Kit. The constructed libraries were quality checked by Agilent Bioanalyzer 2100 and Qubit, and the libraries that passed the quality check were sequenced.

### 2.9 RNA extraction and quantitative real-time polymerase chain reaction analysis

Total RNA was removed from frozen liver tissue using TRIzol reagent (Genes and Bio Co., Ltd., Beijing, China) and was reverse transcribed using Superbrilliant 6 min High-quality RNA Extraction Kit (Zhongshi Gene Technology, Tianjin, China). qRT-PCR was performed using Superbrilliant 2×ZAPA3G SYBR Green qPCR Mix (Zhongshi Gene Technology) on an Applied Biosystems 7500 Fast Real-Time PCR System (Thermo Fisher Scientific, Waltham, MA, USA). Gene-specific primers are listed in **Table 1**.

**Table 1:** Sequences of rat primers used for real-time fluorescence quantitative PCR.

Gene	Sequence (5′-3′)	Length (bp)
*Pdk1*	Forward	ATGTACTCAACTGCACCCCG
	Reverse	TGTGCAGTTACGAGCTTCGG
*Pi3k*	Forward	ACAAAGCTCTACTCTAGGCGTG
	Reverse	TTACCAGCATGGTCATGGGC
*Gsk-3β*	Forward	TCGTCCATCGATGTGTGGTC
	Reverse	TTGTCCAGGGGTGAGCTTTG
*Gs*	Forward	TTGCCAGAATGCACGCAGAA
	Reverse	TGCCTGCATCATCTGTTGAC
*β-actin*	Forward	GATCAGCAAGCAGGAGTACGA
	Reverse	GGTGTAAAACGCAGCTCAGTAAC

### 2.10 Testing hepatic IR-related proteins using western blot

Liver tissue proteins were extracted, and the protein content was measured by the BCA method. Samples were separated by 10% SDS-PAGE and transferred to PVDF membranes. After washing three times with TBST, the membranes were closed with BSA for 2 h. The membranes were washed three times with TBST and incubated with different primary antibodies at 4°C overnight. The next day, the membranes were washed three times with TBST and incubated with secondary antibodies at room temperature for 2 hours at 25°C. The membranes were then washed three times with TBST, and the proteins were detected with extremely ultrasensitive ECL chemiluminescent reagents and quantified by optical densitometry using an image analyzer.

Primary antibodies include rabbit monoclonal antibodies directed against β-actin (1:7000), IRS-1 (1:700), PDK1 (1:1000). Mouse monoclonal antibody against PI3Kp85 (1:5000), P-AKT(1:700) and P-GSK-3β(1:3000). secondary antibody HRP-goat anti-rabbit IgG + HRP-goat anti-mouse IgG (1:8000).

### 2.11 Statistical analysis

All experimental data were statistically analyzed using SPSS Statistics 22.0 software (IBM Corp., Armonk, NY, USA). The data conformed to a normal distribution and were described as mean ± standard deviation. One-way analysis of variance was used for comparisons between multiple groups. The least significant difference test was used if the variance was equal, whereas Tamhane’s test was used if the variance was unequal. *P* < 0.05 was considered statistically significant.

## 3 Results

### 3.1 Quantitative analysis of the active ingredients of TAS

**Figure 1:**
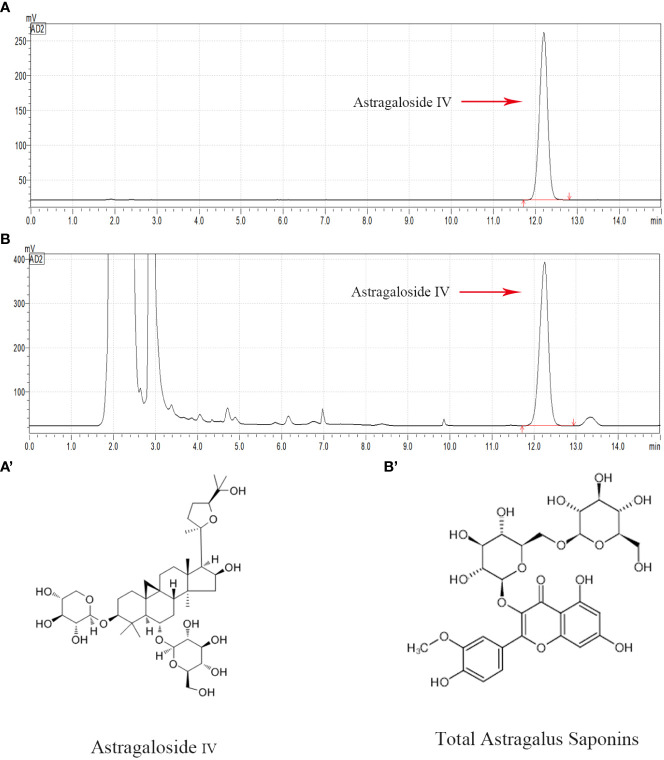
HPLC chromatogram of the TAS aqueous solution. Major compounds of Astragaloside IV in the TAS aqueous solution were identified and compared to the standards by the HPLC method. The tested peaks for Astragaloside IV are indicated with red arrows. **(A)** Astragaloside IV standard; **(B)** Astragaloside IV in TAS; (A') The structural formula of Astragaloside IV; and (B') The structural formula of TAS.

High performance liquid chromatography was used to conduct qualitative and quantitative tests on AS-IV (**Figure 1**), which is a representative component of TAS. Overall, compared with the AS-IV standard substance, a chemical composition test peak of AS-IV was detected in TAS, and this compound accounted for 0.6775 mg/ml of TAS.

### 3.2 Effects of TAS on FBG, PG2h, OGTT-AUC and GSP

**Figure 2:**
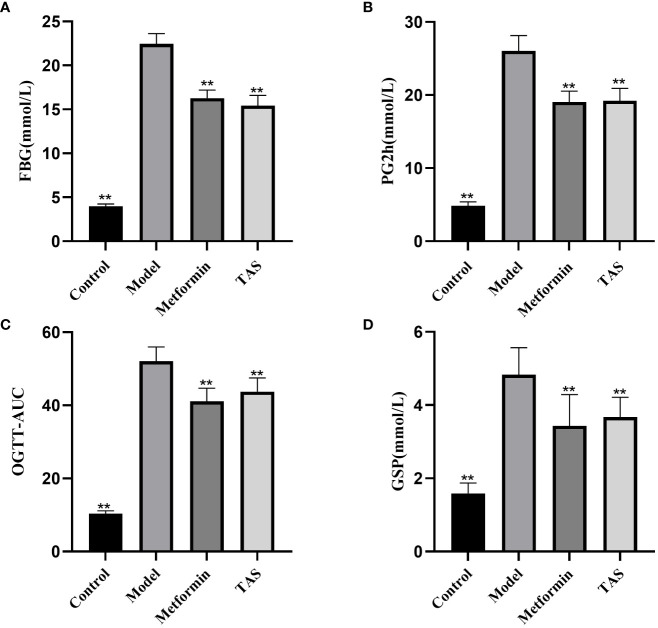
Effects of TAS on fasting blood glucose (FBG), 2 hour postprandial blood glucose (PG2h), oral glucose tolerance tests area under the curve (OGTT-AUC), and glycated serum protein (GSP). **(A)** FBG **(B)** PG2h; **(C)** OGTT-AUC; and **(D)** GSP. **P<0.01 compared with the model group.

As shown in **Figure 2**, the FBG, PG2h, OGTT-AUC, and GSP values were significantly increased in the model group compared with the control group, indicating disordered glucose metabolism and abnormal glucose tolerance in T2DM rats. TAS and metformin decreased the above indexes, indicating that they could improve the abnormal glucose tolerance.

### 3.3 Effects of TAS on INS, C-P and HOMA-IR

**Figure 3:**
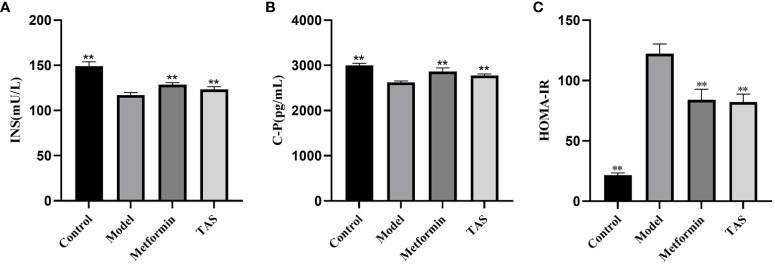
Effects of TAS on serum insulin (INS), C-peptide (C-P) and insulin resistance index (HOMA-IR). **(A)** INS; **(B)** C-P; and **(C)** HOMA-IR. **P<0.01 compared with the model group.

Further analyses revealed that the INS and C-P levels were significantly decreased while HOMA-IR was increased in the T2DM rats, indicating that the function of islet cells was impaired and INS secretion and islet cell sensitivity were reduced (**Figure 3**). Consistent with the above described results, treatment with TAS and metformin significantly increased the serum INS and C-P levels and decreased the HOMA-IR, indicating that TAS can improve the function of islet cells and inhibit IR in T2DM *in vivo*.

### 3.4 Effects of TAS on blood lipid levels

**Figure 4:**
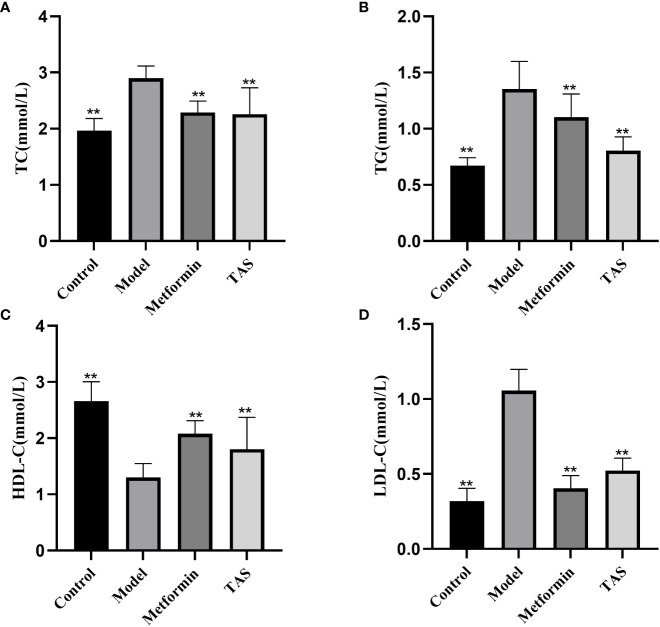
Effects of TAS on serum total cholesterol (TC), triglycerides (TG), low density lipoprotein cholesterol (LDL-C) and high density lipoprotein cholesterol (HDL-C). **(A)** TC; **(B)** TG; **(C)** HDL-C; and **(D)** LDL-C. **P<0.01 compared with the model group.

The TC, TG, and LDL-C levels were also significantly increased in T2DM rats, whereas the HDL-C levels were significantly decreased (**Figure 4**), further demonstrating that the function of islet cells in T2DM rats was impaired, the activity of lipid metabolizing enzymes in the body was decreased, and lipid synthesis was increased. Lipids, such as cholesterol and TG, will deposit in the viscera, thereby causing IR, and the two affect and promote each other. Treatment with either TAS or metformin significantly reduced the TC, TG, and LDL-C levels and increased the HDL-C levels, indicating that TAS can improve the blood lipid metabolism in diabetes.

### 3.5 Effects of TAS on IL-1β and TNF-α

**Figure 5:**
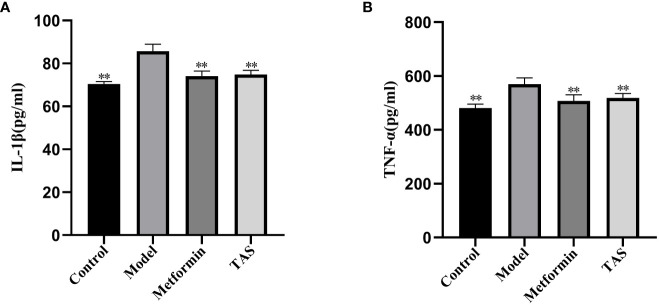
Effects of TAS on serum Interleukin-1β (IL-1β) and tumor necrosis factor-α (TNF-α). **(A)** IL-1β; and **(B)** TNF-α. **P<0.01 compared with the model group.

As shown in **Figure 5**, the levels of IL-1β and TNF-α were significantly higher in the model group compared with the control group, suggesting that increased endotoxins in the circulatory system induced chronic low-level inflammation in T2DM rats. TAS and metformin reduced the levels of related inflammatory factors, suggesting that they could ameliorate this chronic low-level inflammation.

### 3.6 Effects of TAS on liver and colon morphology

**Figure 6:**
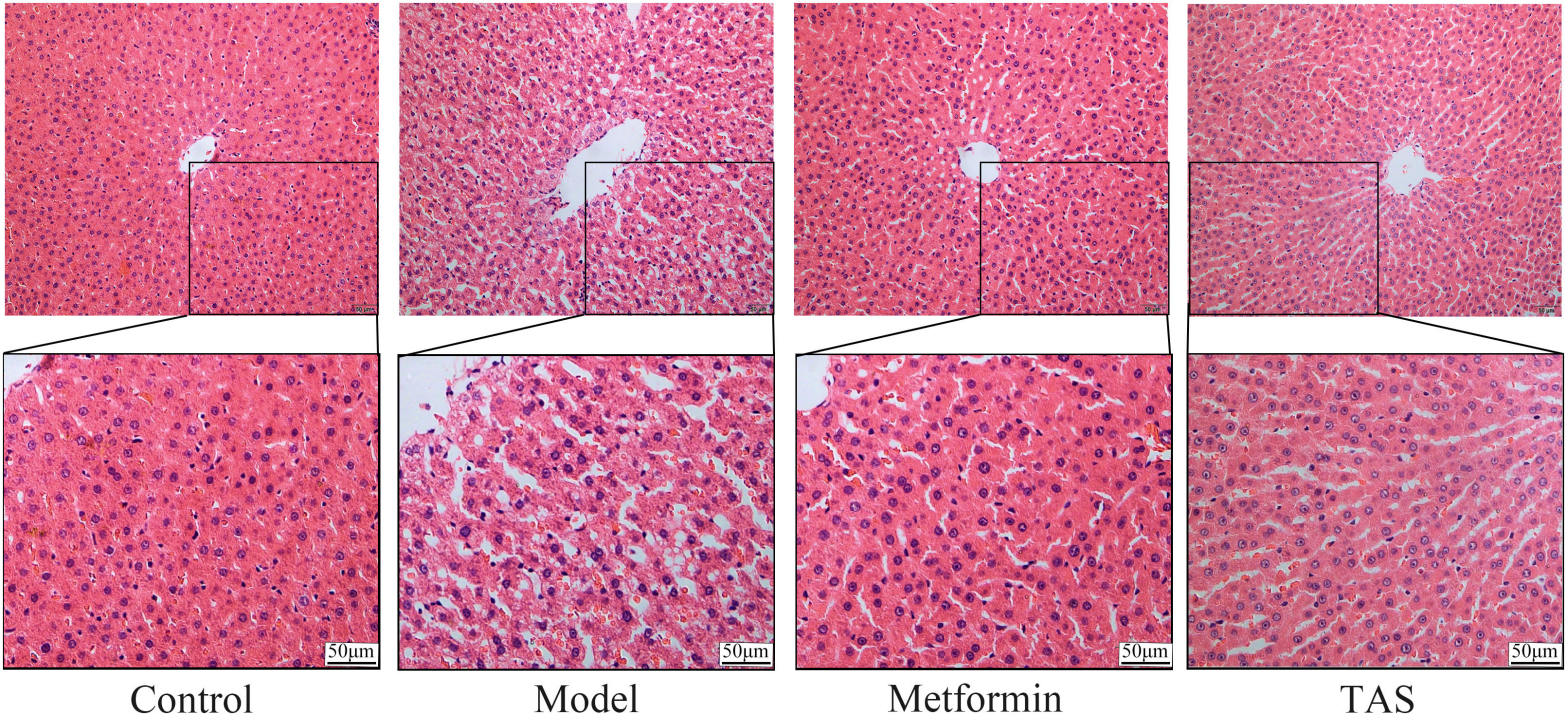
Effects of TAS on the morphology and structure of the liver. Liver tissues were stained with H&E (200×).

HE staining of liver tissue samples collected from the control group showed that the cells presented a close, radial cell-to-cell arrangement, with large round hepatocytes presenting a centered nuclei, abundant cytoplasm, and clear nuclear membrane (**Figure 6**). In contrast, the liver of T2DM rats showed infiltration of inflammatory cells, and the hepatocytes presented a loose arrangement and vacuolated cytoplasm, with steatosis in a small number of hepatocytes. Notably, the liver morphology of T2DM rats improved significantly upon TAS and metformin treatment, with hepatocytes becoming closely arranged together, with occasional observation of steatosis, and presenting an overall form (large, round, with centered nuclei and abundant cytoplasm) similar to that of healthy cells.

**Figure 7:**
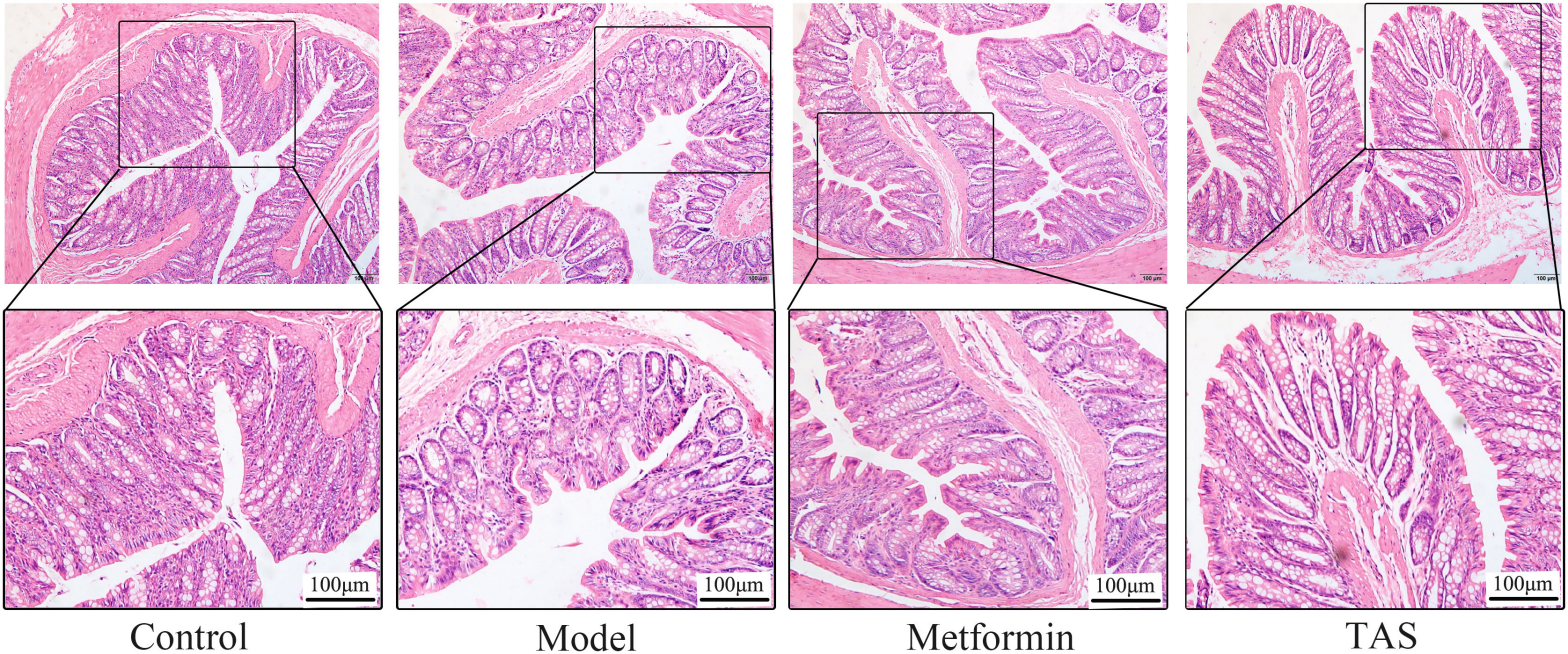
Effects of TAS on the morphology and structure of the colon. Colon tissues were stained with H&E (100×).

HE staining of colon samples from the control group showed that they had an intact mucosal structure with long villi, neatly arranged crypts, and tightly arranged cells. In contrast, despite T2DM rats retaining a basically intact colon mucosal structure with clear crypts and villi, their colon cellular structure was arranged quite loosely and showed infiltration of a small number of inflammatory cells (**Figure 7**). Treatment with metformin and TAS reversed the negative effects of T2DM on the colon tissue, with the mucosal structure of the colon being more complete than that of untreated T2DM rats. Notably, the colon morphology of TAS-treatment rats was basically closer to that of the control animals.

**Figure 8:**
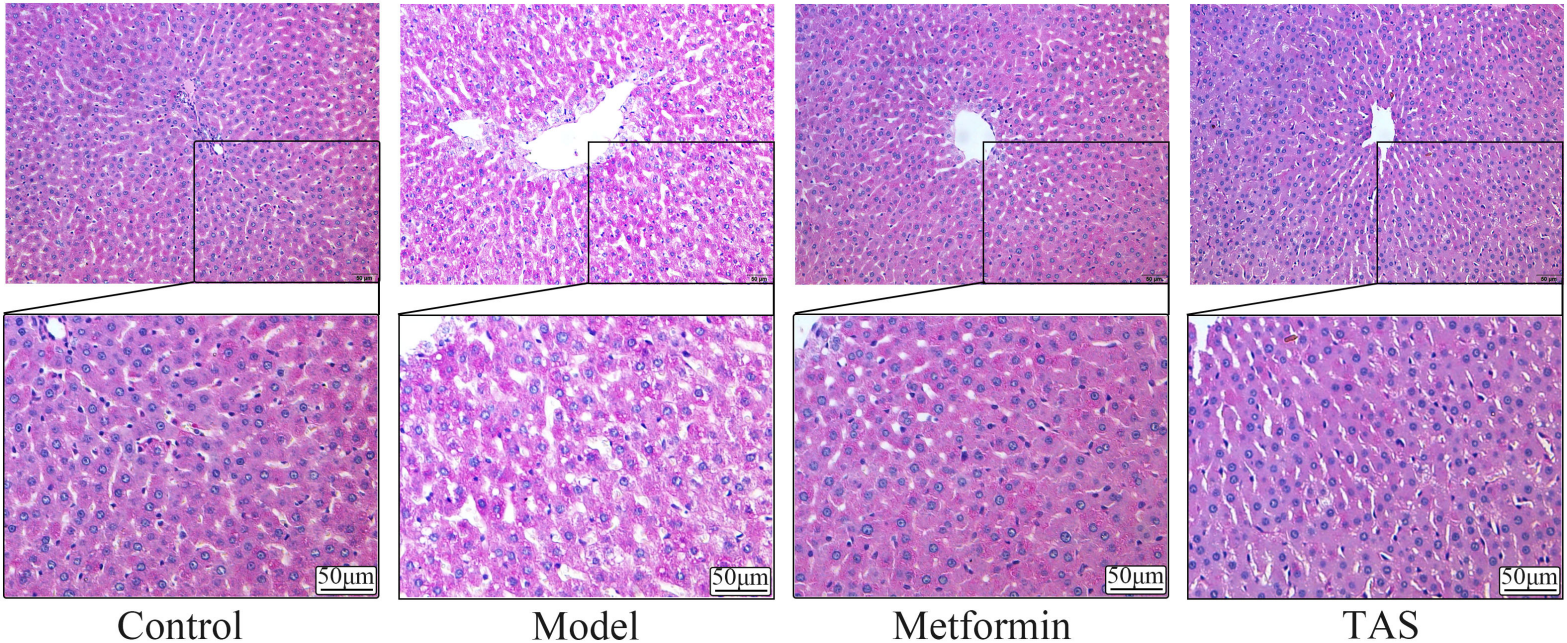
Effects of TAS on the morphology and structure of the liver. Liver tissues were stained with PAS (200×).

As shown in **Figure 8**, full and uniform distribution of liver glycogen was observed in the PAS-stained control group. In contrast, T2DM rats had disordered hepatocyte arrangement, with fat vacuoles, weak glycogen-positive reaction, and sparse and uneven glycogen distribution. Compared with the model group, PAS staining showed a relatively full and homogeneous distribution of glycogen in the TAS and metformin groups, and hepatocyte steatosis was occasionally observed.

### 3.7 Effects of TAS on the intestinal microbiome in T2DM

#### 3.7.1 Effects of TAS on the composition of the intestinal microbiome

**Figure 9:**
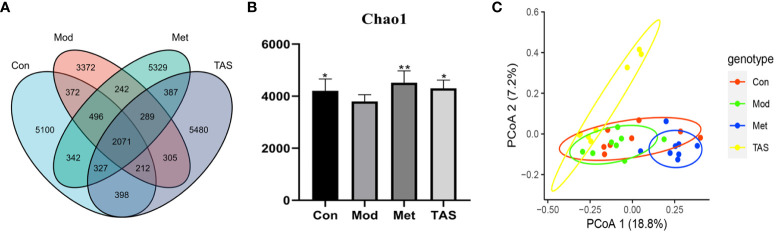
Effects of TAS on the operational taxonomic unit (OTU) clustering and diversity of intestinal bacteria. **(A)** Venn diagram based on OTU distribution. **(B)** Chao1 flora abundance index. **(C)** Principal co-ordinates analysis. ^*^ *P*<0.05, ^**^ *P*<0.01 compared with the model group.

As shown in **Figure 9A**, the 16s intestinal flora assay showed that there were 7359, 9318, 9483, and 9469 species of intestinal flora in the model group, control group, metformin group, and TAS group, respectively. This finding indicates that the number of intestinal flora in T2DM rats was reduced, dysbiosis was significantly improved, and the number of flora was increased after drug administration treatment. Noteworthy, further analysis of the microbial communities showed that the microbiota abundance decreased with T2DM but increased after drug intervention, as demonstrated by changes in the Chao1 index (**Figure 9B**). Principal coordinate analysis was used to investigate the variability of the community composition of the samples, and it further confirmed that the microbial community composition differed significantly between the metformin and TAS-treated groups and model rats (**Figure 9C**). Taken together, these findings demonstrate that TAS can regulate intestinal dysbiosis and restore the intestinal microbiome in T2DM rats.

#### 3.7.2 Effects of TAS on the intestinal microbiome community structure

**Figure 10:**
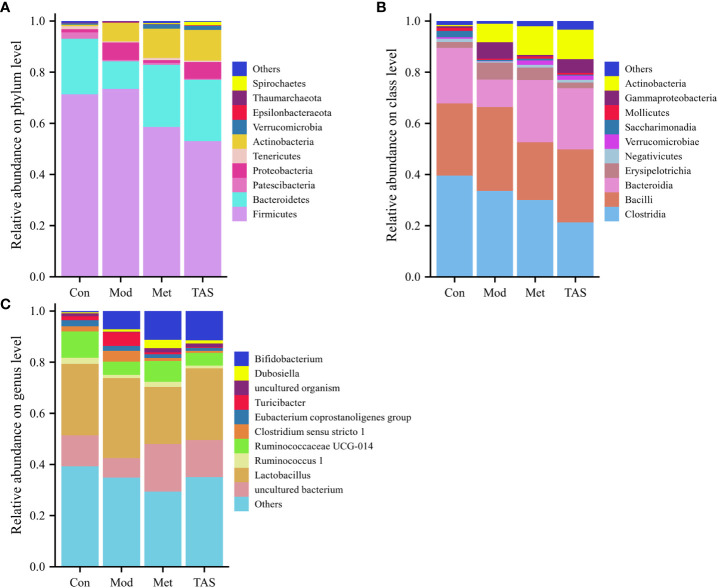
Intestinal bacterial community structure composition of each group of rats. Bacterial taxonomic study at the **(A)** phylum level, **(B)** class level, and **(C)** genus level.

According to the community structure diagram, the types of microorganisms contained in each sample at a certain taxonomic level and the relative abundance of each microorganism in the sample can be visualized. Analysis of the structure of intestinal microbiota at the phylum level yielded 11 phyla, among which Bacteroidetes and Firmicutes dominated. The Firmicutes/Bacteroidetes ratio was 3.28, 6.80, 2.41, and 2.22 in the control, model, metformin, and TAS groups, respectively. Of note, the Firmicutes/Bacteroidetes ratio increased significantly in the T2DM group compared with that in the control group, whereas it decreased significantly in the metformin and TAS groups compared with that in the model rats. The relative decrease in Bacteroidetes in the T2DM group further indicated abnormalities in the intestinal microbiome. After administering metformin and TAS, the proportion of Bacteroidetes in the rat intestine increased while the proportion of Firmicutes decreased and Actinomycetota increased significantly, indicating that metformin and TAS had certain regulatory effects on Bacteroidetes and Actinomycetota, thereby affecting the proportion of the intestinal microbiota (**Figure 10A**).

At the class level, Clostridia, Bacilli, and Bacteroidia were the most common. The control animals had the largest proportion of Clostridia, whereas the T2DM rats showed a decreased proportion of Clostridia and Bacteroidia but an increased proportion of Bacilli. After the administration of TAS and metformin, the proportion of Bacteroidia and Actinomyces increased and the proportion of Bacilli decreased, which is consistent with the above findings at the phylum level (**Figure 10B**).

Lastly, at the genus level, administration of TAS and metformin improved the relative abundance of intestinal microbiota, increased *Bifidobacterium*, *Ruminococcaceae* UCG-014, and uncultured bacterium, and decreasing the relative abundance of *Lactobacillus* and *Turicibacter*. *Bifidobacterium* is an important physiological microbiota in human and animal intestines, known to improve digestive problems and glycemic control and reduce blood lipid levels (23). Therefore, various diseases, such as obesity and diabetes, are associated with a reduced abundance of *Bifidobacterium* (**Figure 10C**).

#### 3.7.3 Effects of TAS on Different Species in Gut Microbiota of T2DM Rats

**Figure 11:**
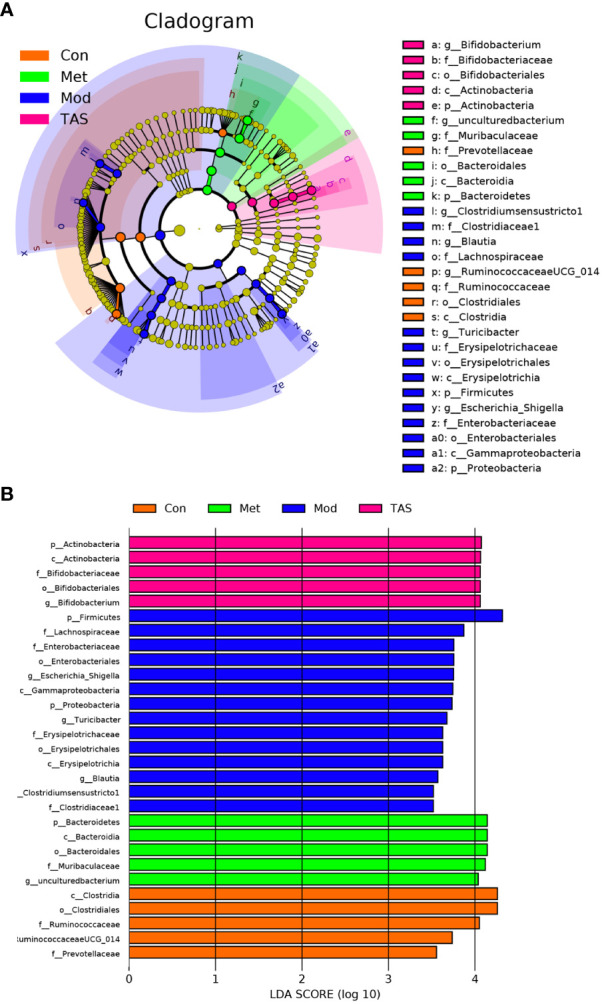
Gut microbiota changes of each group of rats. LEfSe analyses of gut microbiota in the four groups with the LDA scores >3.5. **(A)** Cladogram of the LEfSe analyses. **(B)** Bar graph of LDA scores.

Different bacteria species respond differently to environmental stimuli (24, 25). Linear discriminant analysis effect size (logarithmic discriminant analysis >3.5) was performed on the samples according to different grouping conditions based on the taxonomic composition to identify bacterial communities or species that were significantly and differentially impacted. A total of 29 genera were critical phylotypes and showed significant differences in abundance among the four experimental groups (**Figure 11**). Among them, the model group included g_*Clostridium_sensu_stricto1*, f_*Clostridiaceae1*, g_*Blautia*, f_*Lachnospiraceae*, g_*Turicibacter*, f_*Erysipelotrichaceae*, o_*Erysipelotrichales*, c_*Erysipelotrichia*, p_*Firmicutes*, g_*Escherichia-Shigella*, f_*Enterobacteriaceae*, o_*Enterobacterales*, c_*Gammaproteobacteria*, and p_*Proteobacteria*. The five differential microorganisms in the control group were g_*Ruminococcaceae* UCG-014, f_*Ruminococcaceae*, o_*Clostridiales, c*_*Clostridia*, and f_*Prevotellaceae.* The microorganisms associated with the metformin treatment included p_*Bacteroidetes, o*_*Bacteroidales, c*_*Bacteroidia, f*_*Muribaculaceae*, and g_uncultured bacterium genus. The microorganisms associated with the TAS treatment included p_*Actinobacteria*, c_*Actinobacteria*, o_*Bifidobacteriales*, f_*Bifidobacteriaceae*, and g_*Bifidobacterium.* Bifidobacterium is a representative large group of probiotics in the human intestine that is involved in various physiological functions, such as nutrient acquisition and antibacterial, immunomodulatory, and antitumor effects (26). The above observations suggest that intestinal microbiota in diabetic rats may be remodeled after TAS treatment.

#### 3.7.4 Prediction of the function of intestinal microorganisms regulated by TAS in T2DM

**Figure 12:**
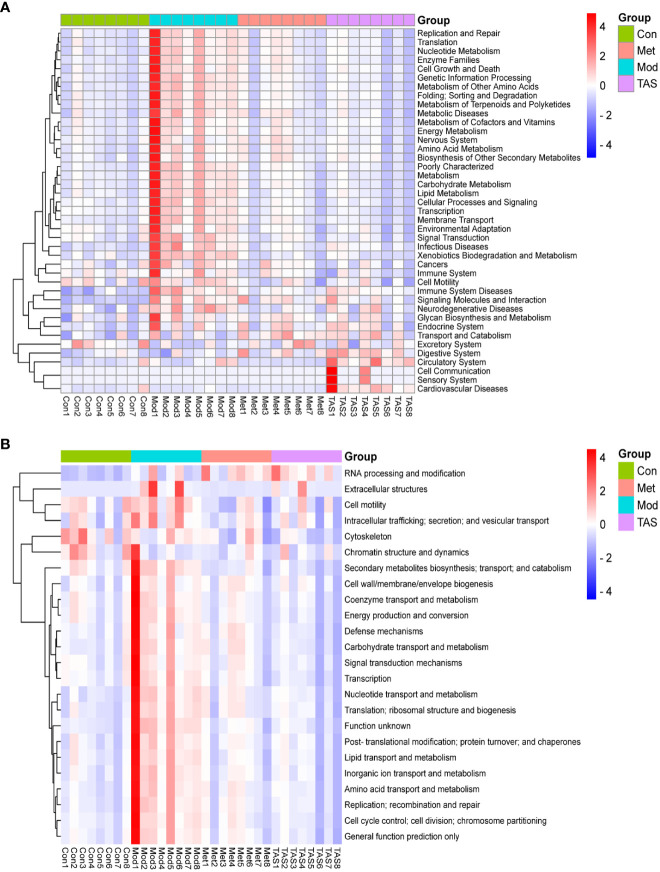
Prediction of intestinal microbial functions in the rats in each group. **(A)** KEGG heatmap analysis. **(B)** COG heatmap analysis.

To further assess the implications of TAS treatment on the intestinal microbiota of T2DM rats, a PICRUSt analysis and 16S rRNA gene sequence data were applied to predict the KEGG pathways (**Figure 12A**) and COG functions (**Figure 12B**) of the bacterial community. Basic metabolic pathways, such as amino acid, carbohydrate, lipid, and energy metabolism, and glycan biosynthesis and metabolism, and pathways associated with the digestive system and endocrine system increased in T2DM rats compared with those in control animals. Moreover, TAS and metformin treatment downregulated these basic pathways metabolisms as compared with those in the model group, although those of the TAS group were more similar to those of the control group. The expression of amino acid, nucleotide, carbohydrate, and lipid transport and metabolism pathways was also increased in the COG functional analysis model group, which was consistent with the KEGG results and more comprehensively revealed the functional composition of the colony. In contrast, the functional expression of the TAS treated group was consistent with that of the control rats. These findings indicate that T2DM caused by a high-fat diet combined with streptozotocin leads to disordered intestinal microbiota. Of note, the TAS treatment improved and even reversed the T2DM-related intestinal microbiome dysbiosis.

### 3.8 TAS improves hepatic IR in rats via the Pi3k/Akt/Gsk-3β pathway

**Figure 13:**
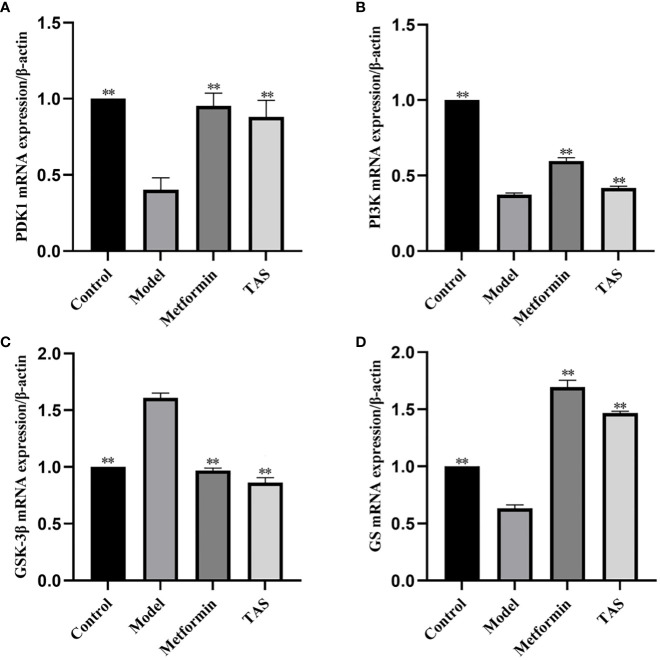
Effect of total astragalus saponins on PDK1, PI3K, GSK-3β, and GS gene expression level. Relative expression of **(A)** PDK1, **(B)** PI3K, **(C)** GSK-3β, and **(D)** GS. **P<0.01 compared with the model group.

The effects of TAS on hepatic phosphoinositide-dependent kinase 1 (*Pdk1*), *Pi3k*, *Gsk3b*, and glycogen synthase 2 (*Gys2*) were evaluated using qRT-PCR based on the predicted functions of the intestinal microbiota (**Figure 13**). Overall, TAS treatment upregulated hepatic *Pdk1*, *Pi3k*, and *Gys2*, and downregulated *Gsk3b* compared with that in the model group. In turn, metformin more significantly upregulated hepatic *Pdk1*, *Pi3k*, and *Gys2* compared with TAS and only slightly downregulated *Gsk3b* compared with TAS.

**Figure 14:**
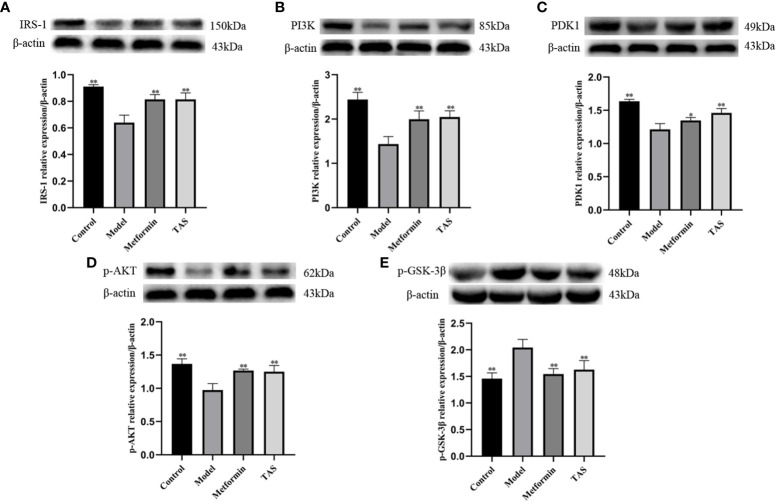
Effect of total astragalus saponins on the expression of insulin resistance related proteins. Relative expression of **(A)** IRS-1 *in vivo*, **(B)** PI3K *in vitro*, **(C)** PDK1 *in vitro*, **(D)** p-AKT *in vivo*, and **(E)** p-GSK-3β *in vivo*. ^*^ *P*<0.05 and ^**^ *P*<0.01 compared with the model group.

Next, western blot analysis of the major proteins of the hepatic Pi3k/Akt/Gsk-3β pathway was performed in TAS treated rats (**Figure 13**). Overall, TAS and metformin were found to effectively increase the levels of INS receptor substrate 1 (Irs-1), Pi3k, and Pdk1, while promoting the phosphorylation of Akt but a reduced expression of phosphorylated Gsk-3β (**Figure 14**).

## 4 Discussion

Studies have shown that TAS has hypoglycemic and hypolipidemic effects (27), but there is little evidence on how TAS modulates gut microbes in T2DM rats. Therefore, the present study investigated the effects of TAS on IR and gut microbes in rats with high-fat and high-sugar diets and STZ-induced diabetes. In this study, the T2DM rats showed significant increases in FBG, PG2h, OGTT-AUC, GSP, and HOMA-IR; significant decreases in INS and C-P levels; significant increases in TC, TG, and LDL-C levels increased significantly; significant decreases in HDL-C levels; and significant increases in IL-1β and TNF-α levels. HE staining of the model group showed inflammatory cell infiltration, cytoplasmic laxity or even vacuolization, fatty degeneration of a few hepatocytes, and degradation of the colonic mucosa structure. PAS staining showed that the liver glycogen content was decreased. These findings indicate that the T2DM rats showed disorders of glucose metabolism and insulin resistance, impaired islet cell function, abnormal lipid metabolism, chronic low-level inflammation, damage to the liver and colon tissues, and decreased liver glycogen content *in vivo*. Notably, treatment with TAS and metformin significantly improved the glucose tolerance, blood lipid metabolism, Inflammatory factor levels, and INS sensitivity of the model rats, improved the morphology and structure of the liver tissues, reduced steatosis of liver tissues, increased the content of liver glycogen; improved the mucosal structure of the colon, and reduced the infiltration of inflammatory cells.

The intestinal microbiome constitutes the largest micro-ecosystem in the human body and is involved in various metabolic processes and energy production (28). In recent years, it has been widely recognized that intestinal microorganisms play important roles in various human diseases and are considered the “second genome” of the human body (29, 30). The intestinal microbiota is characterized by the presence of a large number of genes that metabolize polysaccharides in their genomes (31, 32). Intestinal microbiota can breakdown carbohydrates, which can lead to diabetes when the carbohydrates are not digested or absorbed by the host (33). Studies have demonstrated that dysbiosis can affect the intestinal barrier function, host metabolism, and signal transduction pathways, thereby inducing diabetes (34). A large number of metabolites produced by intestinal microorganisms reach the liver, a central organ involved in glucose homeostasis and diabetes control, through portal circulation. Therefore, the gut-liver axis is an important pathway for regulating hepatic glycolipid metabolisms and has important effects on energy homeostasis (35).

Analysis of the intestinal microbiota in T2DM rats revealed that certain microbiota types as well as the Chao1 index and microbiota abundance were reduced and the structure of the microbial community differed significantly at different taxonomic levels. Among the harmful bacteria, a higher abundance of *Clostridium sensu stricto*, *Blautia*, *Escherichia-Shigella*, and *Proteus* was observed. Studies have found that the abundance of *Bacteroides*, *Escherichia coli*, and *Desulfovibrio* is increased in diabetic patients (36). The abundance of *Bifidobacterium* decreases when blood glucose elevates, which in turn exacerbates diabetes. Herein, *Proteobacteria*, *Escherichia-Shigella*, and *Lachnospiraceae* in T2DM rats were closely associated with hepatic steatosis, inflammatory damage, and fibrosis degree and thus affected lipid metabolism (26, 37). Moreover, *Enterobacter* and Proteobacteria in the T2DM group could potentially disrupt the intestinal micro-ecological environment, leading to increased endotoxins in the host circulatory system and inducing chronic low-level inflammation. This in turn leads to various metabolic disorders (38).

Treatment with TAS and metformin was found to increase the number of bacteria, such as *Bacteroides* and *Bifidobacterium*, regulating dysbiosis and restoring the intestinal microbiota in T2DM. The number of microbiota, Chao1 index, and microbiota abundance increased, and the structure of the community at different taxonomic levels improved upon treatment. Among beneficial bacteria, the abundance of *Ruminococcus*, *Prevotella*, *Bifidobacterium*, *Lactobacillus*, *Lactococcus*, and *Bacteroides* increased. In particular, studies have reported that Ruminococcaceae is closely associated with improved energy metabolism (39). Short-chain fatty acids synthesized with the involvement of *Prevotella* have protective effects on the intestinal mucosal barrier (37). *Bacteroides* can improve high-fat diet-induced metabolic and immune dysfunctions (40); in particular, *Bacteroides acidifaciens* (41) and *Bacteroides uniformis* can improve glucose tolerance and IR (40). Hence, *Bacteroides* plays a beneficial role in glucose metabolism in humans and experimental animals, and its abundance is negatively correlated with T2DM (42, 43). *Muribaculaceae* can degrade dietary and polysaccharides to produce short-chain fatty acids and exhibits anti-inflammatory and glycolipid homeostasis balancing properties (44). *Bifidobacterium* can improve high-fat diet-induced hepatic steatosis and intestinal inflammation, and has several beneficial functions, such as improving liver function, lowering blood lipids, regulating intestinal environment, and anti-aging (45–48). The *Bifidobacterium* level is significantly higher in healthy individuals, and it has a protective activity against T2DM and negatively correlates with T2DM (49–54). This shows that correcting and maintaining the diversity of the gut microbiota is important for improving T2DM. In conclusion, treatment with TAS could improve the composition and community structure of intestinal microbiota by increasing the relative abundance of microbiota and the number of beneficial bacteria, which may be one of the mechanisms by which it improves T2DM.

IR is a critical factor contributing to T2DM (55). Dysregulation of glycogen synthesis or glucogenesis and increased gluconeogenesis caused by hepatic IR are the main causes of fasting hyperglycemia in T2DM patients (56–58). This in turn causes glucotoxicity and impairs islet structure and function (59). INS in the liver binds to the INS receptors tyrosine kinases, phosphorylates IRS-1, and activates the AKT, thereby decreasing hepatic glucose production and promoting glycogen synthesis. In addition, INS increases hepatic glycogen synthesis through glycogen synthase (especially GYS2 in the liver) and regulating regulation glycogen phosphorylase by GSK-3β and protein phosphatase 1 (60, 61). IR observed in T2DM is most likely attributable to a defect in the INS receptor/IRS-1/PI3K/Akt cascade. In this pathway, phosphorylation of Ser/Thr of IRS-1 can inhibit INS-stimulated tyrosine phosphorylation of IRS-1 and has the ability to bind and activate PI3K. The PI3K-p85 protein will generate negative feedback on INS sensitivity upon return. Akt is a major downstream target of PI3K; therefore, its activation leads to inactivation of the GSK-3β-specific isoform, thereby increasing glycogen synthase expression and regulating glucose transport activity (62).

qRT-PCR and western blot analysis confirmed that treatment with TAS increased hepatic Pdk1, Pi3k, and Gys2 expression and decreased Gsk-3β expression, indicating that both upstream and downstream genes of the PI3K/Akt/GSK-3β pathway are activated. The activated upstream gene *PI3K* promotes insulin sensitivity through feedback on IRS-1, whereas the activated downstream genes *glycogen synthase* and *GSK-3β* regulate glucose metabolism by affecting glucose transport and glycogen synthesis. This is consistent with previous findings that AS-IV acts through the PI3K/AKT signaling pathway (15, 63).

There are some limitations to this study. The detailed relationship between gut microbiota still required to be studied. Fecal transplantation and targeted metabolomics may be useful in future to deeply illustrate the metabolic regulatory mechanism of TAS on T2DM based on regulating gut microbiota. Further *in vivo* and *in vitro* experiments will be used to further explore the mechanism of this study in the upcoming studies.

## 5 Conclusion

In summary, the gut-liver axis plays an important role in the development and progression of T2DM. TAS can ameliorate disordered glucose metabolism and lipid metabolism in T2DM, regulate the composition, community structure, and homeostasis of intestinal microbiota, increase the relative abundance and number of *Bifidobacterium*, reverse pathological changes in liver and colon tissues, and improve hepatic INS signal transduction. TAS may have therapeutic potential for T2DM by regulating the intestinal microbiota homeostasis, improving hepatic INS signal transduction, inhibiting gluconeogenesis, and promoting glycogen synthesis. This study provides an experimental basis for TAS to regulate the homeostasis of intestinal flora for the treatment of T2DM, which may represent a new path for guiding the clinical treatment of diabetes.

## Data availability statement

The datasets presented in this study can be found in online repositories. The names of the repository/repositories and accession number(s) can be found in the article/supplementary material.

## Ethics statement

The animal studies were approved by Animal Ethics Committee of North China University of Science and Technology. The studies were conducted in accordance with the local legislation and institutional requirements. Written informed consent was obtained from the owners for the participation of their animals in this study.

## Author contributions

LM, XL, HC, and J-AL conceived and designed the experiments. LM, XL, BZ, WX, MW, and QF conducted the experiments. CT, CW, ZC, HC, and J-AL supervised and advised on the study. LM, XL, HC, and J-AL analyzed the data and wrote the manuscript. All authors approved the final manuscript for publication.
